# Environment, Social, and Governance Performance and Financial Performance With National Pension Fund Investment: Evidence From Korea

**DOI:** 10.3389/fpsyg.2022.893535

**Published:** 2022-05-12

**Authors:** Sungjin Son, Jootae Kim

**Affiliations:** College of Business and Economics, Dankook University, Yong-in, South Korea

**Keywords:** ESG, stewardship code, national pension fund, social performance, carbon emission reduction

## Abstract

This study attempts to examine the relationship between environment, social, and governance (ESG) management and financial performance and the role of socially responsible investment in the National Pension Fund (NPF), Korea’s largest institutional investor. This study tries to provide evidence for the slack resource hypothesis by verifying whether companies with higher financial performance make more efforts to improve ESG performance. In addition, we tried to validate whether NPF is expanding its investments in corporations with high economic performance and high ESG performance. Based on our analysis, Korean companies with good financial performance actively participate in ESG. When we compared the performance between 2019 and 2020, companies with high ESG performance increased regardless of financial performance level, whereas companies with high financial performance and low ESG performance decreased. This represents that the perception and attitude of Korean companies toward ESG management are evolving. NPF has a high investment ratio for firms having a high ratio in both financial and ESG performance. NPF further invested in companies with high ESG performance, even if the financial performance is not decent. This study provides evidence that Korean companies’ interest in ESG management as well as the behavior of socially responsible investment of NPF are rising.

## Introduction

Sustainable management is a long debating issue in business research and is currently understood in three pillars of environment, social, and governance (ESG). ESG is a movement that underscores to maximize corporate financial performance (CFP) and non-financial values including “green practices,” “social and ethical values,” and “improvement of corporate governance” simultaneously.

There have been many attempts to create social values from corporate management even before ESG management was introduced (Bowen, 1953; Friedman, 1970; Carroll, 1991; Kaplan and Norton, 1992; Preston and O’Bannon, 1997; Waddock and Graves, 1997). The international community tried to enhance social value by suggesting “ISO 26000,” “the Paris climate agreement,” “RE100,” and “the EU carbon border tax.” As corporate social responsibility (CSR) becomes important, the role of a corporate citizen to fulfill economic, legal, ethical, and philanthropic responsibilities has also been emphasized (Carroll, 1991).

At present, ESG management becomes more critical than the concept of CSR and encompasses not only social (S) area but also environmental (E) and governance (G) aspects. A company can obtain support from various stakeholders including shareholders and the market by adopting ESG management. The concept of ESG is broader than CSR, and ESG faces stronger regulations. Apple, for example, declared its plan to achieve 100% carbon neutralization in its supply chains and products by 2030, and BlackRock, the world’s largest asset manager from the United States, announced that it would not invest in companies with the sales from thermal power production such as coal (BlackRock, 2020). Additionally, ExxonMobil, the largest US energy company, was excluded from the Dow Jones industrial average.

In Korea, the interest in ESG management is also growing. ESG management is strongly required in Korean companies, especially those operating in global value chains. Within the global value chain, companies must respond to ESG-related requirements in international business environments. The Korean government has started the carbon emission trading market and RE100. Korea Stock Exchange and Financial Transactions Commission have provided ESG-related information such as ESG index, ESG statistics, and domestic/foreign guidelines in the ESG portals (Korea Exchange, 2021; esg.krx.co.kr) since December 2021.

There have been numerous studies to examine how ESG affects CFP. Many studies investigated the relationship between CSR and CFP. Academic papers reporting a positive relationship seem to be dominant (Friede et al., 2015; Sohn, 2016), yet some papers also show a negative relationship (Brammer et al., 2006; Hoepner and McMillan, 2009; Christophe and Viviani, 2015). Slack resource hypothesis can offer the supporting logic for the positive relationship. The relationship between CSR and CFP needs to be further investigated.

Environment, social, and governance requires firms to move from shareholder orientation to stakeholder capitalism and to create social values through various efforts such as reducing greenhouse gas emission, tackling global warming in environmental issues, decreasing racial, or gender discrimination, preventing labor exploitation in social issues, and solving accounting fraud and managerial moral hazard in governance issues. At this point, an inquiry arises to us—what is the aim of ESG? ESG management does not replace shareholder orientation with stakeholder capitalism. The core value pursued by ESG is to maximize not only the financial worth of a company but also the social value made by it. ESG management is consistent with the stakeholder theory of Freeman (1984) and the BSC philosophy of Kaplan and Norton (1992), the slack resource hypothesis of Preston and O’Bannon (1997) and Waddock and Graves (1997). In stakeholder theory and BSC philosophy, it is argued that the enhancement of non-financial performance measures will lead to an increase in economic performance. In the slack resource hypothesis, firms with higher financial performance have more available resources, and they can conduct ESG management more actively to create more social values. The aim of ESG, therefore, should establish the concept of the coexistence of shareholder orientation and stakeholder capitalism. There are many studies that investigated the causal relationship between CSP and CFP, but the question to examine whether social performance is decent in companies with excellent CFP or *vice versa* remains largely unexplored. This study, from the viewpoint of the slack resource hypothesis, aims to investigate if Korean companies with high financial performance tend to show high social performance measured by ESG standards.

In contrast, the role of institutional investors in socially responsible investment becomes significant as the interest in ESG rises. The stewardship code is a part of the UK company law and is a voluntary guideline to actively encourage institutional investors to exercise their voting rights. Korean National Pension Fund (NPF) also began to operate the fund based on socially responsible criteria through the revision of the National Pension Act in January 2015. The legal basis for the fund investment is newly prepared by considering ESG factors.^1^ NPF is the world’s third-largest pension fund with a reserve of KWD 930 trillion (US$84.5 billion) as of 2021. NPF is investing 5% or more shareholding in over 300 companies in the Korean stock market in 2021 (National Pension Fund, 2021). After adopting the stewardship code, NPF exercised shareholder rights actively and considered ESG factors in its investment decision.

As the role of institutional investors in the financial market becomes more important, studies have been conducted that the activism of institutional investors can provide benefits to shareholders (Gillan and Starks, 2000, 2007; Becht et al., 2008; Buchanan et al., 2012; Farooqi et al., 2017; Brav et al., 2018; Routledge, 2020). A general argument in this field is that the activism of institutional investors can provide benefits to shareholders (Becht et al., 2008; Buchanan et al., 2012; Brav et al., 2018). However, there is also opposite evidence to the contrary (Gillan and Starks, 2007; Farooqi et al., 2017; Routledge, 2020). In Korea, NPF declared its investment direction with regard to corporate ESG activities. But there has been less attention to examine whether NPF is strengthening its activism of institutional investors since they adopted the National Pension Fund Responsible Investment Promotion Plan in 2019. This study intends to investigate if NPF, which adopted stewardship code, invests more in corporations with higher social performance measured by ESG standards and whether this NPF’s behavior in ESG investment can show how socially responsible investment is applied in Korea.

The remainder of the study is structured as follows. In the “Theory and Hypotheses” section, we reviewed the literature relevant to our study and developed our hypotheses. The “Methodology” section describes the sample and data. The “Empirical Results” section depicts the research design and explains the results, and the “Conclusion” section offers discussion and conclusion.

## Theory and Hypotheses

### Environment, Social, and Governance Performance and Financial Performance

The aim of ESG management is to remove the dark side caused by achieving the economic prosperity of the current human society and make the human community sustainable for a long time. ESG management performs this function in the pillars of ESG in addition to maximizing a company’s financial performance. There have been attempts to create social value related to ESG even before the ESG concept was introduced. CSR was first mentioned by Bowen (1953) when he defined it as “the duty of entrepreneurs to formulate desirable policies, make decisions, and pursue actions in light of the values and objectives pursued by our society.” Since then, Friedman (1970) and Carroll (1979) tried to define it, and especially Carroll (1991) emphasized the role of a corporate citizen by dividing CSR into four areas, namely, economic responsibility, legal responsibility, ethical responsibility, and philanthropic responsibility.

The international community has recently proposed the Paris climate agreement, RE100, and EU carbon border tax. Global companies started green management to reduce the carbon emission amount and investment institutions such as Blackrock stressed responsible investment.

Recently, ESG-related information disclosure standards were enacted by the Sustainability Accounting Standards Board (SASB) in the United States and the Sustainability Standards Board (SSB) from International Financial Reporting Standards (IFRS) Foundation. Korea followed these trends, and Korea Stock Exchange made it mandatory for listed companies with total assets of over KWD 2 trillion won (US$1.8 billion) to disclose their governance structure in 2019. Additionally, the Ministry of Trade, Industry, and Energy established the K-ESG guidelines in 2021. The Financial Transactions Commission and Korea Stock Exchange provide ESG-related information, such as ESG grades of listed companies, ESG investment product statistics, as well as domestic and foreign guidelines from the ESG portal (esg.krx.co.kr) since December 2021. Along with the efforts of the Korean government, listed companies in Korea are expanding ESG management.

Numerous studies were conducted to examine how the corporate efforts in the areas of ESG affect CFP. The recent papers investigated the relationship between ESG management and CFP. For example, Friede et al. (2015) performed a meta-analysis of 2,200 prior studies about the relationship between ESG and CFP and reported that 48% of the overall sample concluded positive results in the relationship. Sohn (2016) also reported that the relationship between financial characteristics and economic performance of companies that perform CSR in Korean society is positive. However, other studies reported negative, neutral, or mixed relationships (Vance, 1975; Aupperle et al., 1985; Griffin and Mahon, 1997; Wright and Ferris, 1997; Rowley and Berman, 2000; van Beurden and Gössling, 2008; Hoepner and McMillan, 2009; Christophe and Viviani, 2015; Friede et al., 2015; Kwak et al., 2022). Multiple early outcomes about how CSR affects CFP proved a negative relationship (Vance, 1975; Wright and Ferris, 1997). As an early argument, Friedman (1970) asserted that the maximization of shareholders’ profit is the only social responsibility of the corporates. Friede et al. (2015) also reported that 11% of the sample showed the impact of ESG on CFP to be negative, 23% to be neutral, and 18% to be mixed. Kwak et al. (2022) analyzed the sensitivity between fund flow and the performance of Korean funds and whether there would be a difference in the sensitivity between ESG funds and non-ESG funds. The analysis revealed that they had a negative (-) correlation, and the ESG did not influence fund flow. They concluded that investors in Korean ESG funds focused more on non-financial properties rather than on profit. It is recognized that results from the past studies are mixed, and further analysis is still needed.

The most debated question is whether active ESG management of a firm improves its financial performance, or companies with high financial outcomes tend to actively carry out ESG management. There are two opposing arguments for the discussion of the relationship between financial performance and ESG performance. One argument from neoliberalism economists including Friedman (1970) is based on the agency theory. They argued that the management should not engage in actions that undermine shareholder value because the efforts to improve social performance make companies spend corporate resources and undermine short-term corporate value. The other argument, however, is that socially responsible behavior may have a positive effect on corporate financial performance (Friede et al., 2015). This is based on the stakeholder theory started by Freeman (1984) and the slack resource hypothesis asserted by Preston and O’Bannon (1997) and Waddock and Graves (1997). In this dispute, a company with good financial performance is able to increase its activities to enhance social value because it has more available resources.

The ESG management is consistent with the stakeholder theory of Freeman (1984) and the BSC philosophy of Kaplan and Norton (1992), the slack resource hypothesis of Preston and O’Bannon (1997) and Waddock and Graves (1997). So, the core value pursued by ESG should emerge maximizing not only the financial performance of a company but also the social value contrived by it. There have been countless studies to investigate the causal relationship between CSP and CFP, but the question to answer whether social performance is decent in companies with excellent CFP or *vice versa* remains mainly uncharted.

This study, therefore, aimed to examine whether companies with high financial performance make more efforts to improve social performance measured by ESG standards than those with lower financial performance. This assumption is based on the slack resource hypothesis. The first hypothesis is as follows:

***Hypothesis 1:** Korean company with good financial performance has respectable ESG performance.*

### Environment, Social, and Governance-Related Investment of National Pension Fund

The stewardship code was established by the UK Financial Reporting Council in 2010 based on The UK Corporate Governance Code and the ISC Code as part of the UK’s efforts to overcome the financial crisis that occurred in 2008. It is not to regulate companies to improve corporate governance but rather to focus on socially responsible behaviors of institutional investors. The reason is that the financial crisis occurred because institutional investors did not make appropriate investments in firms.

In Korea, NPF was established in 1986 with the vision of contributing to a stable and happy life for people through sustainable pension and welfare services. It also adopted the stewardship code in 2016 to play an important role in corporate governance as an institutional investor. Specifically, NPF prepared a legal basis for accountable investment so that ESG factors are considered for an investment decision. The fund is operated based on the revised National Pension Act in 2015, and the principles of responsible investment were added to the fund management guidelines in 2016. NPF revised Principles on Trusteeship Responsibility (Stewardship Code), which includes the guidelines to exercise shareholder rights, and prepared implementation plans for fiduciary responsibility activities in 2019. NPF added sustainability to the five fund management principles (i.e., profitability, stability, publicity, liquidity, and operational independence) of the NPF’s management guidelines by launching a plan to promote responsible investment in 2019. These efforts have become the basis for NPF and responsible investment, and the exercise of shareholder rights can be continuously promoted (National Pension Fund, 2019).

As the role of institutional investors in the financial market becomes more important, the role of institutional investors in corporate governance is becoming significant from the passive role (Gillan and Starks, 2000). Many prior studies tried to investigate the effective governance role of institutional investors. The general argument is that institutional investor activism can provide benefits to shareholders (Becht et al., 2008; Buchanan et al., 2012; Brav et al., 2018), but there is also opposite evidence (Gillan and Starks, 2007; Farooqi et al., 2017; Routledge, 2020). For example, Mehrani et al. (2017) divided institutional investors into active institutional investors and passive institutional investors, and they reported that active institutional investors had a positive effect on earning quality, but passive institutional investors did not. Routledge (2020) also stated that when internal corporate governance does not properly play its role, responsible investment by institutional investors can serve as an effective external governance structure. However, Gillan and Starks (2007) insisted that institutional investor activism lowers corporate value by hindering managers from pursuing long-term goals. Farooqi et al. (2017) also classified institutional investors into active and passive ones, and after analyzing the effect on corporate credit grade, they reported that the more the passive institutional investors, the higher the corporate credit score.

Some Korean literature analyzed NPF as an institutional investor. Kim et al. (2015) investigated the accounting characteristics of companies in which NPF acquired over 5% of shareholding from 2010 to 2013. It was shown that companies in which NPF acquired a large number of shares had higher performance in profitability [ROA and return on equity (ROE)] and growth (net profit growth rate). NPF prefers stocks with a high price-earning ratio (PER). This is evidence supporting that NPF invests more in companies with excellent profitability, growth potential, and stock price return. Meanwhile, Kim and An (2018) studied the relationship between the percentage of shareholding of NPF and CSR activities. They found that there was a positive relationship between the ownership ratio of NPF and CSR activities. Firms in which NPF holds over 5% shares for 3 years are more progressive in CSR activities. This may be evidence that NPF is playing a successful monitoring role as an institutional investor.

Unlike CSR, ESG is stressed by investors. Investors utilize a company’s ESG scores for their investment decision.^2^ To achieve ESG-related investment, institutional investors should reduce a negative externality caused by market imperfections and management activities. Institutional investors must enhance profitability in the long-term perspective. It is necessary to reduce market imperfections and an externality from management activities (Richardson, 2007). In Korea, NPF declared its investment strategy with regard to the ESG activities of firms. However, less attention has been paid to examine if NPF is strengthening its activism of institutional investors ever since they adopted National Pension Fund Responsible Investment Promotion Plan in 2019. This study, therefore, tries to examine whether NPF, which has implemented the stewardship code to protect the wealth of the investors in the fund, invests in companies with higher social performance and higher financial performance. Additionally, this study aims to provide evidence on how socially responsible investment has prevailed in Korea. The second hypothesis is as follows:

***Hypothesis 2:** A company with higher financial and ESG performance will have a larger percentage of shareholding by Korean NPF.*

## Methodology

### Data Collection

The variables in this study are financial performance, ESG management index, and ownership rate of NPF. Financial performance represents the economic position in a specific period of a company from financial statements. Although various existing indicators show financial performance, this study selects the ROE as a proxy,^3^ which shows how much profit the invested equity capital has generated. ESG management index refers to the degree to which a company performs desirable activities in terms of ESG. There are diverse ESG indices both domestically and globally that measure the level of ESG management, and all these indices have pros and cons. This study uses the ESG score published by Korea Corporate Governance Service (KCGS). KCGS evaluates ESG management by seven grades (i.e., S, A+, A, B+, B, C, and D). NPF discloses the ownership rate of the invested firm. This study utilizes the percentage of shareholding revealed by NPF.

### Sample Selection

We obtained ESG data from the KCGS’s ESG index for 2019 and 2020. NPF adopted the stewardship code in 2018. It began to exercise shareholder rights for the purpose of preventing agency problems by large shareholders. In 2019, NPF adopted a plan to promote responsible investment, and since then, NPF considers ESG factors for the investment decision to play the governance role as the Korea’s largest institutional investor. The purpose of this study was to inspect the relationship among financial performance, ESG performance, and ownership rate of NPF for Korean companies for the period 2019 and 2020. This analysis is valuable because ESG investment started to be emphasized during the study period of this research in Korea. Financial data of the companies were attracted from Fn-guide, and the ownership rate of NPF was collected from NPF disclosure on its website. The sample was collected as shown in Table 1.

**TABLE 1:** Sample selection.

	2019		2020		Total
			
	Remove	*N*	Remove	*N*	*N*
1. ESG grade of KCGS from ESG portal	–	963	–	1,005	1,968
2. Companies without ESG sub-grades	55	908	55	950	1,858
3. Companies not included in Fn-guide, and companies in capital impairment	6	902	5	945	1,847
4. Total		902		945	1,847

We collected ESG data for 1,968 companies from the KCGS’s ESG index reported for 2019 and 2020. Fifty-five companies were excluded because some scores were omitted. Eleven companies were removed because they had the impairment of the capital or did not have the financial performance data in Fn-guide. The final sample was 902 firms in 2019 and 945 firms in 2020. The total number of the sample is 1,847 company years. The industries in our sample are presented in Table 2, which is based on the Korean standard industry classification used on Korea Stock Exchange.

**TABLE 2:** Distribution of sample by industry.

(A) Distribution in the entire sample			(B) Distribution in manufacturing industry		
	
Industry	*N*	%	Industry	*N*	%
Agriculture, fishing, mining	10	0.5	Food and beverage	83	7.5
Manufacturing	1,112	60.2	Textile and clothing	47	4.2
Distribution	136	7.4	Paper and wood	40	3.6
Construction	59	3.2	Chemistry	226	20.3
Transportation, warehousing	50	2.7	Pharmaceuticals	137	12.3
Information and communication	131	7.1	Non-metallic minerals	52	4.7
Financial insurance	121	6.6	Steel metal	96	8.6
Service	228	12.3	Machinery	115	10.3
Total	1,847	100.0	Electrical and electronic	156	14.0
			Medical	18	1.6
			Transportation equipment	112	10.1
			Other manufacturing	30	2.7
			Total	1,112	100.0

Panel (A) shows the distribution of the sample by industry. The manufacturing industry accounted for 60.2%, which is the highest proportion, followed by the service industry, distribution industry, and information and communication industry. In panel (B), among the manufacturing industries, the portion of the subindustries is presented. Chemicals (226, 20.3%), electrical and electronic (156, 14.0%), pharmaceuticals (137, 12.3%), machinery (115, 10.3%), and transportation equipment (112, 10.1%) account for relatively high portions. The distribution of the sample according to the ESG grades by each year for 1,847 samples is shown in Table 3.

**TABLE 3:** Environment, social, and governance (ESG) performance.

(A) Distribution by ESG grade								

2019	S	A +	A	B +	B	C	D	Total
	0	14	92	147	319	303	27	902
	253 (28.1%)				649 (71.9%)			

2020	**S**	**A +**	**A**	**B +**	**B**	**C**	**D**	**Total**

	0	14	179	156	280	296	20	945
	349 (36.9%)				597 (63.1%)			

**(B) E, S, G distribution for samples with ESG grade B + or higher**												

**2019**	**E**				**S**				**G**			
			
	**E > B +**	**B**	**C**	**D**	**S > B +**	**B**	**C**	**D**	**G > B +**	**B**	**C**	**D**

	150	73	22	8	226	27	0	0	230	23	0	0
	59.3%	40.7%			89.3%	10.7%			90.9%	9.1%		
	253 (100.0%)				253 (100.0%)				253 (100.0%)			

**2020**	**E**				**S**				**G**			
			
	**E > B +**	**B**	**C**	**D**	**S > B +**	**B**	**C**	**D**	**G > B +**	**B**	**C**	**D**

	243	79	22	5	328	20	0	1	334	15	0	0
	69.6%	30.4%			94.0%	6.0%			95.7%	4.3%		
	349 (100.0%)				349 (100.0%)				349 (100.0%)			

Panel (A) shows the distribution of the ESG index. In 2019, 253 companies with B + or higher grades were 28.1%, and 649 (71.9%) companies have B or lower grades. In 2020, 349 (36.9%) and 597 (63.1%) were classified respectively. Panel (B) presents the individual scores of ESG for companies with an ESG index of B + or higher. Interestingly, some companies with an ESG index of B + or higher have low scores in each area of ESG. Especially, the scores in E are relatively low. In 2019, 103 (40.7%) from 253 companies did not obtain high scores in E, and in 2020, 106 (30.4%) from 349 companies are in the same case. In Korea, the Green New Deal policy was only introduced in 2020 and during the years 2019 and 2020, and Korean firms’ performance in E was relatively poor compared with their performance in S or G.

## Empirical Results

### Results of Hypothesis 1

**FIGURE 1:**
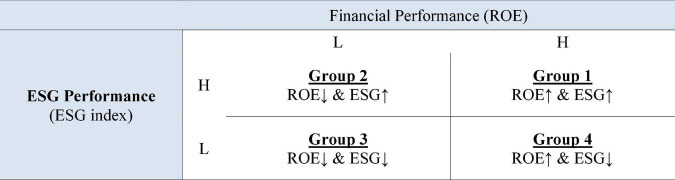
Group classification by financial performance [return on equity (ROE)] and environment, social, and governance (ESG) performance.

In hypothesis 1, we examined whether Korean companies with high financial performance have high ESG performance at the same time. The criteria for judging high or low ROE was referred to Fiegenbaum (1990) and Min and Kim (2019). The two groups were classified based on the median ROE. If the ROE was above the 60th percentile, it was classified as a company with good financial performance, and if it fell below the 40th percentile, it was classified as a company with bad financial performance. ESG performance was grouped based on the ESG index developed by KCGS. If the index is above or equal to B+, the company was considered to have decent ESG performance. We could create a 2 × 2 matrix based on ROE and ESG index as shown in Figure 1.

To test hypothesis 1, we used the chi-square test and difference test of population ratio. Independence verification was tested to examine the significance of the difference in the frequency of high and low ESG performances and high and low financial performances (ROE). Then, if the group 1 (G1) has a higher frequency than the group 2 (G2), the result of the difference test of population ratio is statistically significant (G1 > G2), or the group 4 (G4) has a lower frequency than the group 3 (G3), and it is statistically significant (G4 < G3), it is supported that a firm with good financial performance actively conducts ESG management.

The results of the chi-square test and the difference test of population ratio for all industries in this study are presented in Table 4.

**TABLE 4:** Chi-square test and difference of population ratio test for all industries.

(A) Sample distribution by ROE level						
	60th Percentile	40th Percentile	Total	Median (ROE_19_ = 4.35)		Total
2019	ROE_19_ ≥ 5.83	ROE_19_ ≤ 2.23		ROE_19_ ≥ 4.35	ROE_19_ < 4.35	
	352 (48.8%)	369 (51.2%)	721	442 (49.0%)	460 (51.0%)	902
	60th Percentile	40th Percentile	Total	Median (ROE_20_ = 4.08)		Total
2020	ROE_20_ ≥ 6.03	ROE_20_ ≤ 2.39		ROE_20_ ≥ 4.08	ROE_20_ < 4.08	
	385 (50.8%)	373 (49.2%)	758	480 (50.6%)	465 (49.4%)	948
Year Pooled	Good FP	Bad FP	Total			1,847
	737 (49.8%)	742 (50.2%)	1,479			

**(B) Chi-square test for four groups made by ROE and ESG index**			

Classification	Bad FP (ROE_19_ ≤ 2.23, ROE_20_ ≤ 2.39)	Good FP (ROE_19_ ≥ 5.83, ROE_20_ ≥ 6.03)	Total
Good EP (ESG ≥ B +)	G2 184 (12.4%)	G1 271 (18.3%)	455 (30.8%)
Bad EP (ESG ≤ B)	G3 558 (37.7%)	G4 466 (31.5%)	1,024 (69.2%)
Total	742 (50.2%)	737 (49.8%)	1,479 (100.0%)
Chi-square test	Degree	Pearson Chi-Square	*p*-value
	1	24.884	0.01
			

**(C) Financial performance by ESG performance (ESG) - difference of population ratio test**			

**Good EP (ESG ≥ B +)**		**Bad EP (ESG ≤ B)**	
	
**Good FP**	**Bad FP**	**Good FP**	**Bad FP**

G1 271 (18.3%)	G2 184 (12.4%)	G4 466 (31.5%)	G3 558 (37.7%)
Difference of population ratio test (H_0_: p_1_-p_2_ = 0)		Difference of population ratio test (H_0_: p_1_-p_2_ = 0)	
*Z* = 4.078		*Z* = 2.875	
Two-tail test *p*-value < 0.01		Two-tail test *p*-value < 0.01	

*1. G1: ROE↑, ESG↑, G2: ROE↓, ESG↑, G3: ROE↓, ESG↓, G4: ROE↑, ESG↓, FP, financial performance; SP, ESG performance.*

Panel (A) shows the distribution of the ROE level for all industries. For the 2019 sample, the median of ROE_19_ is 4.35, companies above the 60th percentile (ROE_19_ ≥ 5.83) were classified as having decent financial performance, and companies below the 40th percentile (ROE_19_ ≤ 2.23) were classified as having poor financial performance. Notably, 181 from 902 companies were eliminated and 721 companies remained. Among them, 352 (48.8%) firms have good financial performance, and 369 (51.2%) firms have bad financial performance. From the sample of the year 2020, 385 companies (50.8%) were grouped as having respectable financial performance and 373 companies (49.2%) having unsatisfactory financial performance. In total, 737 (49.8%) companies with good financial performance and 742 (50.2%) companies with bad financial performance were included in the year-pooled data of this study. With a sample of 1,479 firm-year, we tested hypothesis 1.

The results of the chi-square test for the four groups are presented in panel (B). Among 737 companies with decent financial performance, 271 companies with good ESG performance and 466 companies with bad ESG performance exist. Among 742 companies with poor financial performance, 184 companies with good ESG performance and 558 companies with bad ESG performance were found. From the chi-square test for the four groups above, the statistics of Pearson chi-square was 24.884, and the *p*-value was 0.01, which means that the null hypothesis is rejected. There is a statistically significant difference between the high and low levels of financial performance (ROE) and ESG performance. Panel (C) shows the result of the difference test of parent ratios for distribution of a 2 × 2 matrix produced by financial performance and ESG performance. Among the 455 companies with good ESG performance, companies with good financial performance (G1) were 271 (18.3%), and companies with bad financial performance (G2) were 184 (12.4%). Additionally, it was statistically significant with a *Z*-value of 4.078 and a *p*-value of 0.01. Among 1,024 companies with bad ESG performance, companies with bad financial performance (G3) were 558 (37.7%), companies with good financial performance (G4) were 466 (31.5%), and the difference is also statistically significant.

In summary, the frequency of companies with respectable financial performance (ROE) and ESG performance is greater than those with indecent ROE and good ESG (G1 > G2). The frequency of companies with good ROE but with bad ESG was smaller than those with bad ROE and bad ESG (G4 < G3). We conclude that hypothesis 1 in our study is supported. To control the differences by industries, we analyzed it again for the only manufacturing industry, which accounted for 60.2% of the sample.^4^ The result is shown in Table 5.

**TABLE 5:** Chi-square test and difference of population ratio test for the manufacturing industry.

(A) Sample distribution by ROE level						
	60th Percentile	40th Percentile	Total	Median (ROE_19_ = 3.28)		Total
2019	ROE_19_ ≥ 5.10	ROE_19_ ≤ 1.77		ROE_19_ ≥ 3.28	ROE_19_ < 3.28	
	207 (48.1%)	223 (51.9%)	430	262 (48.5%)	278 (51.5%)	540
	60th Percentile	40th Percentile	Total	Median (ROE_20_ = 3.64)		Total
2020	ROE_20_ ≥ 5.13	ROE_20_ ≤ 2.04		ROE_20_ ≥ 3.64	ROE_20_ < 3.64	
	237 (51.4%)	224 (48.6%)	461	293 (51.2%)	279 (48.8%)	572
Year Pooled	Good FP	Bad FP	Total			1,112
	444 (49.8%)	447 (50.2%)	891			

**(B) Chi-square test for four groups made by ROE and ESG index**			

Classification	Bad FP ROE_19_ ≤ 1.77, ROE_20_ ≤ 2.04	Good FP ROE_19_ ≥ 5.10, ROE_20_ ≥ 5.13	Total
Good SP (ESG ≥ B +)	G2 105 (11.8%)	G1 144 (16.2%)	249 (27.9%)
Bad SP (ESG ≤ B)	G3 342 (38.4%)	G4 300 (33.7%)	642 (72.1%)
Total	444 (49.8%)	447 (50.2%)	891 (100.0%)
Chi-square test	Degree	Pearson Chi-Square	*p*-value
	1	8.846	0.01

**(C) Financial performance by ESG performance - difference of population ratio test**			

**Good SP (ESG ≥ B +)**		**Bad SP (ESG ≤ B)**	
	
**Good FP**	**Bad FP**	**Good FP**	**Bad FP**

G1 144 (16.2%)	G2 105 (11.8%)	G4 300 (33.7%)	G3 342 (38.4%)
Difference of population ratio test (H_0_: p_1_-p_2_ = 0)		Difference of population ratio test (H_0_: p_1_-p_2_ = 0)	
*Z* = 2.471		*Z* = 1.657	
Two-tail test *p*-value < 0.01		Two-tail test *p*-value < 0.05	

*1. G1: ROE↑, ESG↑, G2: ROE↓, ESG↑, G3: ROE↓, ESG↓, G4: ROE↑, ESG↓, FP, financial performance; EP, ESG performance.*

The distribution of the ROE level in the manufacturing industry is presented in panel (A). Two groups were made by the criteria of above the 60th percentile of ROE and below the 40th percentile. Notably, 444 companies (49.8%) have good financial performance, and 447 companies have poor financial performance. Of 1,112 companies, 221 were removed, and the remaining 891 companies were tested.

The results of the chi-square test for four groups are given in panel (B). From the chi-square test, the statistic of Pearson chi-square was 8.846, and the *p*-value was 0.01, so the null hypothesis was rejected. This represents that there is a difference between the high and low levels of financial performance (ROE) and social performance (ESG). The difference test of parent ratios for the distribution of financial performance and ESG performance is summarized in panel (C). Among the 249 companies with good ESG performance, companies with good financial performance (G1) are 144 (16.2%), and companies with bad financial performance (G2) are 105 (11.8%). The difference is statistically significant with a *Z*-value of 2.471 and a *p*-value of 0.01. Among 649 companies with bad ESG performance, the number of companies with bad financial performance (G3) is 342 (38.4%), companies with good financial performance (G4) are 300 (33.7%), and the difference is also statistically significant. The analysis of the manufacturing industry showed that ESG management is active in companies with excellent financial performance like the results from all industries.

Then, we tested the relationship between financial performance and the performance in each area of ESG, E, S, and G the using chi-square test and difference test of population ratio. The results for the difference in the population ratio are summarized in Table 6.

**TABLE 6:** Difference of population ratio test for ESG and financial performance.

(A) All industries				(B) Manufacturing industry			
	
(A)-1 ESG (E)				(B)-1 ESG (E)			
	
ESG(E) Good (ESG ≥ B +)		ESG(E) Bad (ESG ≤ B)		ESG(E) Good (ESG ≥ B +)		ESG(E) Bad (ESG ≤ B)	
			
Good FP	Bad FP	Good FP	Bad FP	Good FP	Bad FP	Good FP	Bad FP
G1 185 (12.5%)	G2 134 (18.1%)	G4 552 (37.3%)	G3 608 (41.1%)	G1 111 (12.5%)	G2 91 (10.2%)	G4 333 (37.4%)	G3 356 (40.0%)
*Z* = 2.855		*Z* = 1.645		*Z* = 1.407		*Z* = 0.876	
Two-tail, *p*-value < 0.01		Two-tail, *p*-value < 0.05		Two-tail, *p*-value < 0.10		Two-tail, *p*-value < 0.10	

**(A)-2 ESG (S)**				**(B)-2 ESG (S)**			
	
**ESG(S) Good (ESG ≥ B +)**		**ESG(S) Bad (ESG ≤ B)**		**ESG(S) Good (ESG ≥ B +)**		**ESG(S) Bad (ESG ≤ B)**	
			
**Good FP**	**Bad FP**	**Good FP**	**Bad FP**	**Good FP**	**Bad FP**	**Good FP**	**Bad FP**

G1 344 (23.3%)	G2 231 (15.6%)	G4 393 (26.6%)	G3 511 (34.6%)	G1 176 (19.8%)	G2 122 (13.7%)	G4 268 (30.1%)	G3 325 (36.5%)
*Z* = 4.712		*Z* = 3.924		*Z* = 3.128		*Z* = 2.341	
Two-tail, *p*-value < 0.01		Two-tail, *p*-value < 0.01		Two-tail, *p*-value < 0.01		Two-tail, *p*-value < 0.01	

**(A)-3 ESG (G)**				**(B)-3 ESG (G)**			
	
**ESG(G) Good (ESG ≥ B +)**		**ESG(G) Bad (ESG ≤ B)**		**ESG(G) Good (ESG ≥ B +)**		**ESG(G) Bad (ESG ≤ B)**	
			
**Good FP**	**Bad FP**	**Good FP**	**Bad FP**	**Good FP**	**Bad FP**	**Good FP**	**Bad FP**

G1 409 (27.7%)	G2 335 (22.7%)	G4 328 (22.2%)	G3 407 (27.5%)	G1 231 (23.9%)	G2 189 (21.2%)	G4 213 (23.9%)	G3 258 (29.0%)
*Z* = 2.712		*Z* = 2.913		*Z* = 2.049		*Z* = 2.073	
Two-tail, *p*-value < 0.01		Two-tail, *p*-value < 0.01		Two-tail, *p*-value < 0.01		Two-tail, *p*-value < 0.05	

*1. G1: ROE↑, ESG↑, G2: ROE↓, ESG↑, G3: ROE↓, ESG↓, G4: ROE↑, ESG↓, FP, financial performance; EP, ESG performance.*

Panel (A) shows the result of the analysis for all industries, and Panel (B) shows the result for the only manufacturing industry. First, the analysis for the performance in E among ESG is shown in panel (A)-1 and panel (B)-1, respectively. As a result, G1 has a greater frequency than G2, and G4 has a lower frequency than G3. Then, the analysis of the performance in social and governance areas is found in panels (A)-2 and (B)-2 and in panels (A)-3 and (B)-3. All results are similar to those from the previous analysis, where the total ESG score is considered.

**FIGURE 2:**
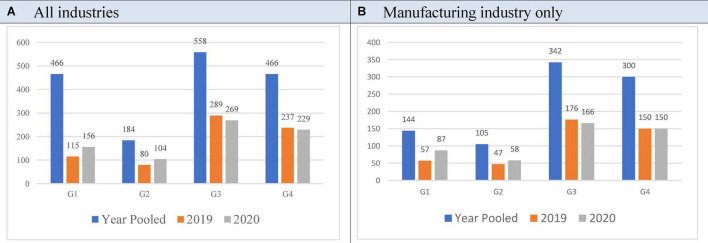
Distribution of four groups (G1, G2, G3, and G4). *^a^*G1: ROE↑, ESG↑, G2: ROE↓, ESG↑, G3: ROE↓, ESG↓, G4: ROE↑, ESG↓.

Additionally, the chi-square test and the difference test of population ratio are performed for the years 2019 and 2020 each. This analysis can compare the result of hypothesis 1 for two different years. This is in Figure 2.

Panel (A) shows the result of the analysis for all industries. The outcome of the analysis for each of the 2 years is the same as the results from 2 years in total. In each of the 2 years, companies with good financial performance also show decent ESG performance (G1 > G2, G4 < G3). A finding is that when comparing the trends from 2019 to 2020, G1 and G2 are increasing, and G4 and G3 are decreasing. Panel (B) shows the result of the analysis for the only manufacturing industry. The result is the same as that for entire industries. From the analysis, even if ESG management in Korea is in its early stage, the interest in ESG management and the corresponding corporate actions are improving.

### Results of Hypothesis 2

In hypothesis 2, we tested whether a company with high financial and ESG performance will have a larger percentage of shareholding by NPF. We compared the shareholding rates of NPF in four groups created by high and low levels of financial performance (ROE) and ESG performance. Analysis of variance (ANOVA) and *post hoc* analysis were performed to test hypothesis 2. The output from the analysis for entire industries is found in Table 7.

**TABLE 7:** ANOVA and *post hoc* analysis for all industries.

(A) ANOVA, independent variable = percentage of NPF									

Classification	*N* (= 1,479)	Mean	*SD*		SS	Degree	MS	*F*-value	*p*-value
G1	271	6.10	4.48	WG	5051.63	3	1683.87		
G2	184	4.65	4.24					151.64	0.01
G3	558	1.16	2.26	BG	16381.84	1475	11.11		
G4	466	2.55	3.20						
Levene static = 121.88 *p*-value = 0.01									

**(B) *Post hoc* table**				

	**G(I)**	**G(J)**	**MD(I-J)**	***p*** **-value**

Dunnett T3	1	2 3 4	1.44 4.92 3.55	0.01 0.01 0.01
	2	3 4	3.48 2.09	0.01 0.01
	3	4	−1.39	0.01

*1. G1: ROE↑, ESG↑, G2: ROE↓, ESG↑, G3: ROE↓, ESG↓, G4: ROE↑, ESG↓.*

Panel (A) shows the ANOVA results, with G1 having the highest average of 6.10, followed by G2 (= 4.65), G4 (= 2.55), and G3 (= 1.16). However, the Levene statistic was 121.88, and the *p*-value was 0.01, indicating heteroscedasticity among groups. Therefore, Dunnett T3 analysis was added to solve the heteroscedasticity problem and validate the differences among groups, and the result is presented in panel (B) and shows that G1 had a larger shareholding rate of NPF than G2, G3, and G4, and the difference was also significant at the 1% level. G2 had a larger percentage of shareholding of NPF than G3 and G4. G4 shows a larger rate than G3. The results of the ANOVA and the *post hoc* analysis for the manufacturing industry are found in Table 8.

**TABLE 8:** ANOVA and *post hoc* analysis for the manufacturing industry.

(A) ANOVA, dependent variable = percentage of NPF									

Classification	*N* (= 891)	Mean	*SD*		SS	Degree	MS	*F*-value	*p*-value
G1	144	6.09	4.43	BG	2925.63	3	975.21		
G2	105	4.31	4.31					96.60	0.01
G3	342	1.00	1.98	WG	8954.01	887	10.10		
G4	300	2.49	3.09						

Levene static = 92.889, *p*-value = 0.01.				

**(B) *Post hoc* table**				

	**G(I)**	**G(J)**	**MD(I-J)**	***p*** **-value**

		2	1.78	0.01
	1	3	5.09	0.01
Dunnett T3		4	3.60	0.01
	2	3	3.31	0.01
		4	1.82	0.01
	3	4	−1.49	0.01

*1. G1: ROE↑, ESG↑, G2: ROE↓, ESG↑, G3: ROE↓, ESG↓, G4: ROE↑, ESG↓.*

From the examination, NPF has a larger shareholding rate in G1 than in G2, G3, and G4. This is similar to the results for entire industries. We also found the same result when comparing G2 with G3 and G4 and G3 with G4. ANOVA and *post hoc* analysis are performed for three areas of ESG, and the result is in Table 9.

**TABLE 9:** ANOVA and *post hoc* analysis for ESG and financial performance.

(A) All industries									(B) Manufacturing industry only								
	
(A)-1 ESG (E)									(B)-1 ESG (E)								
	
	*N* (= 1,479)	Mean	*F*	*p*	G(I)	G(J)	I-J	*p*		*N* (= 891)	Mean	*F*	*p*	G(I)	G(J)	I-J	*p*
G1 G2 G3 G4	185 134 608 552	6.86 5.06 1.36 2.84	148.53	0.01	1	2 3 4	1.80 5.50 4.02	0.01 0.01 0.01	G1 G2 G3 G4	111 91 356 333	6.41 4.41 1.11 2.74	87.06	0.01	1	2 3 4	2.00 5.30 3.67	0.01 0.01 0.01
					2	3 4	3.70 2.22	0.01 0.01						2	3 4	3.30 1.67	0.01 0.01
					3	4	−1.48	0.01						3	4	−1.63	0.01

**(A)-2 ESG (S)**									**(B)-2 ESG (S)**								
	
	***N*** **(= 1,479)**	**Mean**	***F***	***p***	**G(I)**	**G(J)**	**I-J**	***p***		***N*** **(= 891)**	**Mean**	***F***	***p***	**G(I)**	**G(J)**	**I-J**	***p***

G1 G2 G3 G4	344 231 511 393	5.54 4.06 1.11 2.38	128.81	0.01	1	2 3 4	1.48 4.43 3.16	0.01 0.01 0.01	G1 G2 G3 G4	176 122 325 268	5.66 3.93 0.97 2.34	88.60	0.01	1	2 3 4	1.73 4.69 3.32	0.01 0.01 0.01
					2	3 4	2.95 1.68	0.01 0.01						2	3 4	2.96 1.59	0.01 0.01
					3	4	−1.27	0.01						3	4	−1.37	0.01

**(A)-3 ESG (G)**									**(B)-3 ESG (G)**								
	
	***N*** **(= 1,479)**	**Mean**	***F***	***p***	**G(I)**	**G(J)**	**I-J**	***p***		***N*** **(= 891)**	**Mean**	***F***	***p***	**G(I)**	**G(J)**	**I-J**	***p***

G1 G2 G3 G4	409 335 407 328	5.03 3.14 1.11 2.38	88.48	0.01	1	2 3 4	1.89 3.92 2.65	0.01 0.01 0.01	G1 G2 G3 G4	231 189 258 213	4.72 2.84 1.00 2.51	49.34	0.01	1	2 3 4	1.88 3.72 2.21	0.01 0.01 0.01
					2	3 4	2.03 0.76	0.01 0.05						2	3 4	1.84 0.33	0.01 0.92
					3	4	−1.27	0.01						3	4	−1.51	0.01

*1. G1: ROE↑, ESG↑, G2: ROE↓, ESG↑, G3: ROE↓, ESG↓, G4: ROE↑, ESG↓.*

Panel (A) is created by analyzing all industries, and Panel (B) is the result of analyzing the only manufacturing industry. First, the level of social performance was considered in E only, and the results are in Panel (A)-1 and Panel (B)-1. G1 has a larger percentage of shareholding of NPF than other groups (G2, G3, and G4). G2 had a larger percentage of shareholding of NPF than G3 and G4, and G4 also shows a larger ratio than G3. However, in the analysis of the only manufacturing industry when G is considered for social performance, there is no statistical significance between G2 and G4.

As a result, the analysis for hypothesis 2 is summarized as follows. First, G1 has a larger percentage of shareholding by NPF than other groups (G1 > G2, G1 > G3, G1 > G4), and NPF is seen to invest more in companies with respectable financial and ESG performance. Second, G2 has a larger percentage of shareholding by NPF than G3. If the financial performance is low, NPF tends to invest more in companies with decent ESG performance. Third, G4 has a larger percentage of shareholding by NPF than G3. NPF seems to invest more in companies with favorable financial performance if ESG activity is insufficient. Fourth, G2 has a higher percentage of shareholding by NPF than G4, and NPF invests more in companies with satisfactory ESG performance rather than in those with good financial performance. This may be the evidence that Korean NPF stresses ESG performance more than financial performance for its investment decision, but more studies are required in the future.

Interestingly, G is considered as social performance, and there is no difference in NPF investment between high financially performing firms and low financially performing firms. When E or S is considered, the result is different. Even if financial performance is not entirely satisfactory, NPF invests more in companies with exceptional social performance in E or S. NPF may not consider governance factors as seriously as environmental or social components.

## Conclusion

This study investigated the relationship between financial performance and ESG performance, along with the shareholding ratio of NPF for listed companies in Korea. From the analysis of hypothesis 1, it is found that companies with favorable financial performance are more active in ESG management. From the comparison between the years 2019 and 2020, G1 and G2 with decent ESG performance are increasing, and G4 and G3 with low ESG performance are decreasing. This shows that even if ESG management in Korea is in its early stage, social interest and corporate participation are improving.

In hypothesis 2, NPF has more shareholding in companies with decent financial performance and ESG performance. NPF is shown to invest more in companies with respectable ESG performance rather than in those with good financial performance. This is consistent with the fact that pension funds in the world are interested in ESG management in addition to financial output and that sustainability management becomes important to attract investment from outside institutions. For NPF, the fiduciary responsibility for the Korean people is critical, and stable profitability should be kept in the fund operation. NPF cannot pursue public interests such as ESG at the expense of stability and profitability of the fund as a pension fund for all Korean people. The basic purpose of NPF is to create a firm revenue from the investment to guarantee Korean people’s old-age income.

Even if financial performance is not superb, NPF invested more in companies with respectable performance in E or S areas but not in G. Corporate governance is still a critical issue in Korean companies, especially for Korean chaebol firms. The dispute about governance reform in Korea became active after the currency crisis in 1997. Approximately 20 years have passed since the crisis, but the agency difficulties caused by the owner-managers of Korean chaebol firms should be studied further. There has been much dispute about E issues such as climate change since Korean Green New Deal was announced in July 2020, and most discussions about ESG management are in the area of carbon emission reduction. Corporate governance reform in Korea remains for future research.

This study attempted to investigate the relationship between ESG management and financial performance and the role of socially responsible investment in NPF. Additionally, we discovered another evidence to hitherto discussion for the slack resource hypothesis that companies with good social performance can have decent financial performance and confirmed that NPF is starting to strengthen its activism of institutional investors. The main contribution of this study is to show that interest in ESG management is increasing, and the investment considering ESG is also expanding in Korea.

This research has the following limitations.

First, from the analysis of hypothesis 1, companies with good financial performance have decent ESG performance. However, this does not represent a causal relationship between the two variables. We cannot argue that improvement in ESG performance leads to better financial performance or *vice versa*. Even if social performance has improved through ESG management, there may be a time lag in order to lead to the increase in the financial performance since financial performance improvement comes from complex interactions of various factors such as macroeconomic/microeconomic E and internal/external circumstances of the company. It would be helpful to consider a time series research method to solve this limitation. The study period is from 2019 to 2020 when Korean NPF just began to take interest in ESG investment. Given the brief period of research and the early stage of ESG management in Korea, it is hard to design a time-series method for our analysis. Moreover, the year 2020 was a very unusual economic circumstance due to the COVID-19 pandemic, and the results of this study might be affected by the event. However, we could not control the epidemic by designing separate experiments before and after the pandemic due to the inherent limitation of the research duration of this study. In the future examination, we can extend the study term, analyze the causal relationship between financial performance and ESG performance, and explore moderating effects of shareholding of NPF in the relationship between CSP and CFP. Second, from the analysis of hypothesis 2, we found that NPF has performed the role of institutional investor activism by enlarging its investment in companies with respectable social performance rather than those with good financial performance. However, it was not clear from our analysis whether the institutional activism of Korean NPF is only a temporary trend in the early ESG stage or will it continue. Third, it is found that NPF did not make an investment in companies with favorable financial and G performance. It may be a sign to require further investigation of the weak Korean governance structure in Korean chaebols.

## Data Availability Statement

The raw data supporting the conclusions of this article will be made available by the authors, without undue reservation.

## Author Contributions

JK: research design, hypotheses development, and theory building. SS: data analysis and hypotheses development. Both authors contributed to the article and approved the submitted version.

## Conflict of Interest

The authors declare that the research was conducted in the absence of any commercial or financial relationships that could be construed as a potential conflict of interest.

## Publisher’s Note

All claims expressed in this article are solely those of the authors and do not necessarily represent those of their affiliated organizations, or those of the publisher, the editors and the reviewers. Any product that may be evaluated in this article, or claim that may be made by its manufacturer, is not guaranteed or endorsed by the publisher.

## Footnotes

1 A provision for responsible investment was added in the fund management guidelines in 2016. NPS laid the groundwork for exercising shareholder rights by enacting and revising the Principles on Trusteeship Responsibility (Stewardship Code) and related guidelines in 2018, and NPF has tried to raise the shareholder value of investment companies through the adoption of the Stewardship Code. NPF adopted National Pension Fund Responsible Investment Promotion Plan in 2019 and added “sustainability” in addition to “profitability,” “stability,” “publicity,” “liquidity,” and “operational independence.” Since then, NPF decided to consider not only financial components but also environmental (E), social (S), and governance (G) factors.

2 According to the result of the survey conducted by Morgan Stanley (2019), most asset managers (79%) surveyed were putting financial considerations at the fore front of their sustainable investing strategies. Additionally, 82% suggested that strong ESG practices can lead to higher profitability, and those companies with such practices may be better in the long-term investments.

3 In this study, we selected ROE as a proxy of financial performance because ROE can show management efficiency of capital as it measures owner’s equity only excluding debt. We, of course, could choose ROA instead of ROE. In that case, we should consider that the weighted average cost of capital may be lowered due to the tax reduction effect, and thus, the enterprise value may increase. We also should acknowledge that rise in the debt-to-equity ratio may advance the risk of bankruptcy and diminish the corporate value. In addition, above all, society cannot require companies to implement ESG activities using debt. Therefore, we suggested that ROE is a more suitable indicator to exam the effects of ESG activities on CFP.

4 In addition, this study solely analyzed the manufacturing industry which has large samples. We, unfortunately, could not analyze our hypotheses for other industries due to their small sample size. It will be a future research opportunity.
